# Atrial Flutter as an Initial Presentation of Malignant Pericardial Effusion Caused by Lung Cancer

**DOI:** 10.7759/cureus.11712

**Published:** 2020-11-25

**Authors:** Kulachanya Suwanwongse, Nehad Shabarek

**Affiliations:** 1 Internal Medicine, Lincoln Medical Center, New York City, USA

**Keywords:** atrial flutter, cardiac arrythmia, pericardial effusion, dyspnea, lung cancer, case report

## Abstract

Pericardial effusion is a common cardiac condition that can be lethal if left untreated. Patients who have pericardial effusion often present with dyspnea, chest discomfort, chest tightness, and cough. Cardiac arrhythmia as an initial presentation of pericardial effusion is not common. While the most common cardiac arrhythmia associated with pericardial effusion is atrial fibrillation, atrial flutter as a presenting symptom of malignant pericardial effusion has been described rarely. Herein, we present the case of an elderly man who had developed atrial flutter due to malignant cardiac effusion, which subsequently led to the diagnosis of lung cancer. Clinicians should broaden the differential diagnosis of patients who present with atrial flutter. Also, point-of-care ultrasound (POCUS) may help determine the etiology of a new-onset atrial flutter.

## Introduction

Pericardial effusion is defined as an abnormal accumulation of fluid in the pericardial sac, which can result from various etiologies, including inflammation, infection, metabolic, traumatic, and malignancy. Common clinical presentations of pericardial effusion include dyspnea, chest discomfort, chest tightness, and cough. Cardiac arrhythmia is an uncommon clinical presentation of pericardial effusion, while atrial fibrillation is the most common cardiac arrhythmia associated with this condition [1,2]. Herein, we present a unique case of an elderly man who came to our hospital due to atrial flutter from malignant pericardial effusion caused by newly diagnosed lung cancer.

This case report was presented as a poster at the 2020 CHEST annual meeting (virtual) on October 18, 2020.

## Case presentation

**Figure 1:**
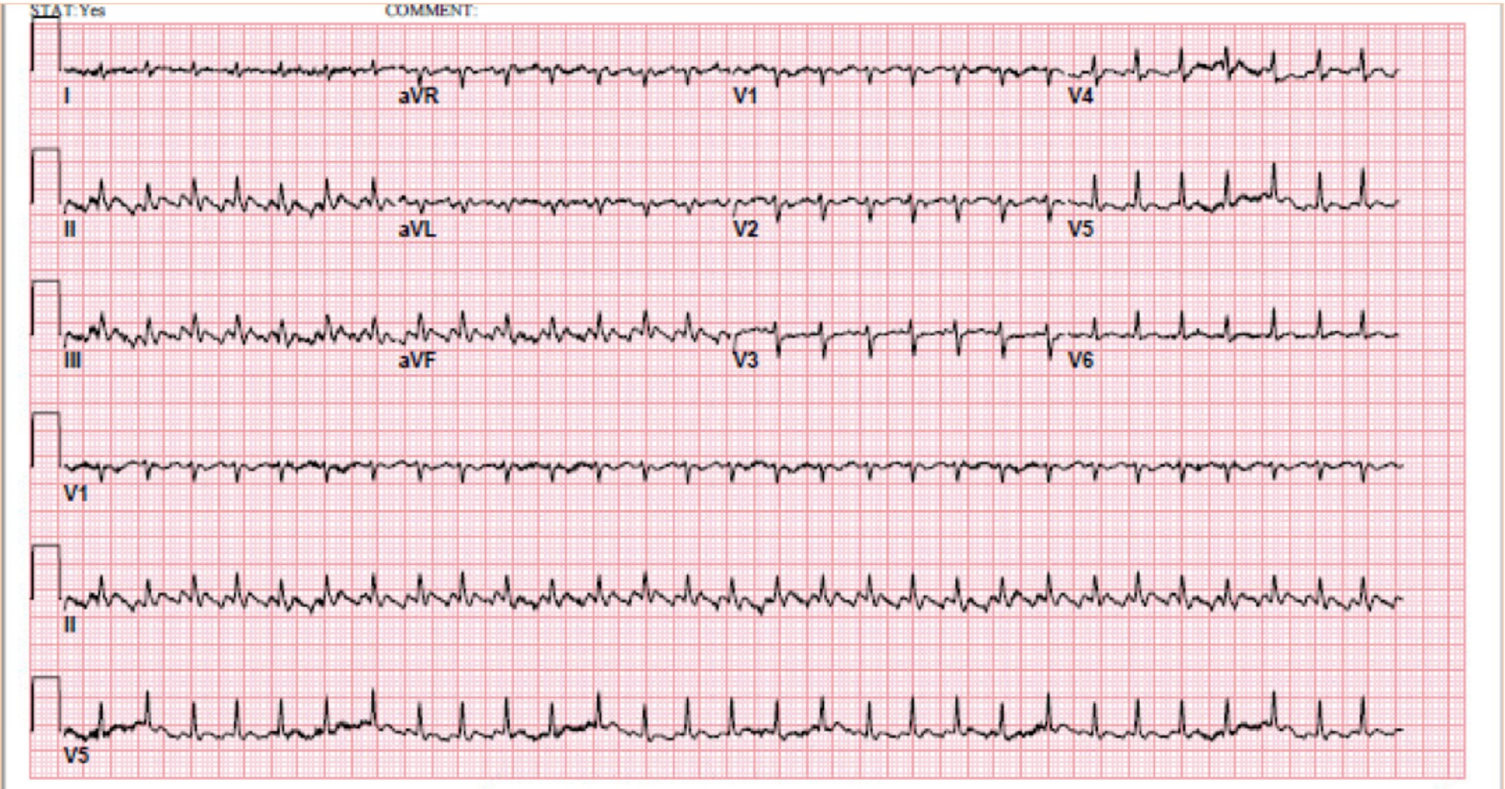
electrocardiogram showing atrial flutter characterized by a rapid ventricular rate and 2:1 atrioventricular block

An 81-year-old man, who was an ex-smoker, presented to our emergency department with acute onset of shortness of breath, non-productive cough, and swelling of bilateral lower extremities. His past medical history was significant for chronic obstructive pulmonary disease and hypertension. He used an albuterol metered-dose inhaler prior to the presentation but his symptoms did not improve. On initial evaluation, the patient had a heart rate of 179 beats per minute (bpm), blood pressure of 172/118 mmHg without pulsus paradoxus, and a respiratory rate of 26 per minute. He had good mental status and was speaking full sentences. There was no jugular venous distention. His lung exam was normal. A heart exam revealed tachycardia without murmur. He had mild bilateral pitting edema of both legs without signs of infection or inflammation. His blood test was unremarkable. Initial ECG (electrocardiogram) showed atrial flutter characterized by a rapid ventricular rate and 2:1 atrioventricular block as demonstrated in Figure 1.

**Figure 2:**
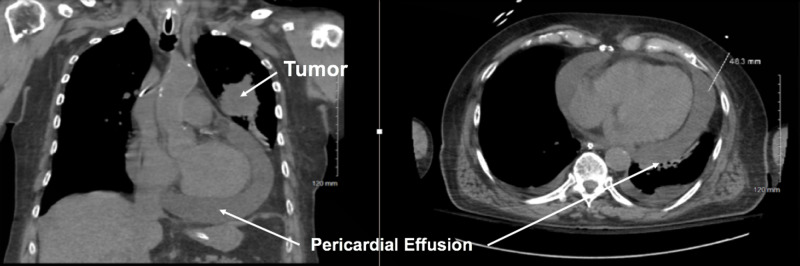
chest CT showing large pericardial effusion and a mass in the upper left lung

We administered a 20 mg bolus of diltiazem to the patient intravenously and his heart rate decreased to 90 bpm. Point-of-care ultrasound (POCUS) showed a large pericardial effusion without ultrasonographic signs of tamponade or signs of pulmonary edema. Computed tomography (CT) of the chest illustrated the large pericardial effusion and led to the discovery of a new mass in the upper left lung, consistent with a lung tumor (Figure 2). Several hours later, while awaiting admission, he became tachypneic and was intubated due to impending acute respiratory failure, despite negative tamponade physiology on previous imaging. The interventional radiologist performed pericardial drainage. Cytology from the pericardial effusion revealed squamous cell carcinoma. The patient was diagnosed with malignant pericardial effusion caused by advanced-stage squamous cell lung cancer. Unfortunately, his hospital course was complicated by septic shock, and eventually, death.

## Discussion

Lung cancer is the most common cause of metastasis to the heart and pericardium because of the proximity of the tumor to the heart [3]. There are several possible explanations for malignant pericardial effusion. Malignant pericardial effusion can be a result of the occlusion of the cardiac lymphatic drainage caused by the tumor [4]. Local or hematogenous spread of the tumor can lead to the obstruction of venous blood flow, resulting in an increase in hydrostatic pressure thus causing pericardial effusion [5]. Besides, neoplastic pericardial involvement stimulates an overproduction of pericardial fluid [5]. In addition, pericardial effusion in patients with malignancy may arise as a complication of the treatment, including chemotherapy and radiation, and hypoalbuminemia from malignancy-related cachexia.

Clinical manifestations of malignant pericardial effusion are similar to pericardial effusion from any other etiology, which include chest discomfort, shortness of breath, tachycardia, cardiac tamponade, shock, and death. The severity of symptoms depends on the rate of fluid accumulation rather than the actual amount of fluid.

Atrial flutter as the first presentation of pericardial effusion is rare. Previous research found 9 out of 789 patients with pericardial metastasis had developed atrial arrhythmias (atrial fibrillation and atrial flutter) [2]. Our case also highlighted the beneficial role of POCUS in determining the underlying causes of atrial flutter. This may encourage the practical use of POCUS in patients with new-onset atrial flutter, especially those with uncertain etiologies.

Cytological analysis of pericardial fluid and pericardial biopsy should be performed in suspected patients to confirm the diagnosis of malignant pericardial effusion. A malignant pericardial effusion is a negative prognostic factor associated with an increase in mortality [1]. Management options in patients with malignant pericardial effusion are pericardiocentesis, pericardial window, pericardial sclerosis, and radiation therapy.

## Conclusions

We presented a rare case of atrial flutter secondary to malignant pericardial effusion, which subsequently led to the diagnosis of advanced-stage lung cancer. Malignant pericardial effusion may occur due to direct extension of the tumor, or due to its lymphatic or hematogenous spread. The diagnosis is confirmed by cytological analysis of the pericardial fluid and pericardial biopsy. Treatment options include pericardiocentesis, pericardial window, pericardial sclerosis, and radiation therapy.

## References

[1] Press OW, Livingston R (1987). Management of malignant pericardial effusion and tamponade. JAMA.

[2] Krisanda TJ (1990). Atrial fibrillation with cardiac tamponade as the initial manifestation of malignant pericarditis. Am J Emerg Med.

[3] DeCamp Jr MM, Mentzer SJ, Swanson SJ, Sugarbaker DJ (1997). Malignant effusive disease of the pleura and pericardium. Chest.

[4] Chiles C, Woodard PK, Gutierrez FR, Link KM (2011). Metastatic involvement of the heart and pericardium: CT and MR imaging. Radiographics.

[5] Scheinin SA, Sosa-Herrera J (2014). Cardiac tamponade resembling an acute myocardial infarction as the initial manifestation of metastatic pericardial adenocarcinoma. Methodist Debakey Cardiovasc J.

